# Modelling and optimizing a system for testing electronic circuit boards

**DOI:** 10.1186/s40929-017-0012-0

**Published:** 2017-09-20

**Authors:** Stephen Y. Chen, Odile Marcotte, Mario Leonardo Morfin Ramírez, Mary Pugh

**Affiliations:** 1 School of Information Technology, York University, 3068 TEL Building, 4700 Keele Street, Toronto, M3J 1P3 Canada; 2 GERAD, HEC Montréal and Département d’informatique, UQAM, 3000 Côte-Sainte-Catherine, Montréal, H3T 2A7 Canada; 3 MOAI Solutions Inc., 3-143 Arlington Ave., Toronto, M6C 2Z3 Canada; 4 Department of mathematics, University of Toronto, 40 St. George St, room 6290, Toronto, M5S 2E4 Canada

**Keywords:** Electronic circuit board, Combinatorial optimization, Covering, Mixed integer programming, Travelling salesman problem

## Abstract

In this article we consider a difficult combinatorial optimization problem arising from the operation of a system for testing electronic circuit boards (ECB). This problem was proposed to us by a company that makes a system for testing ECBs and is looking for an efficient way of planning the tests on any given ECB. Because of its difficulty, we first split the problem into a covering subproblem and a sequencing subproblem. We also give a global formulation of the test planning problem. Then we present and discuss results pertaining to the covering and sequencing subproblems. These results demonstrate that their solution yields testing plans that are much better than those currently used by the company. Finally we conclude our article by outlining avenues for future research.

## Introduction

In this article we study the operations of a system for testing Electronic Circuit Boards (ECBs) that uses flying probes. Our study was carried out in cooperation with a company that will be referred to as *the Company* in what follows. We first describe the broad outlines of this system (called the *FP system*); further details will be given in the following sections. The FP system includes eight *shuttles*: four shuttles that are above the board and four shuttles that are below the board. To each shuttle are attached some *probes* (usually two or three probes). The board being tested contains *nets*, each of which can be viewed as a wire with a finite number of *points of interest* (or simply points). For the purposes of this study, a net is a set of points whose coordinates are known. Note that the nets are pairwise disjoint, i.e., no point belongs to more than one net.

**Fig. 1:**
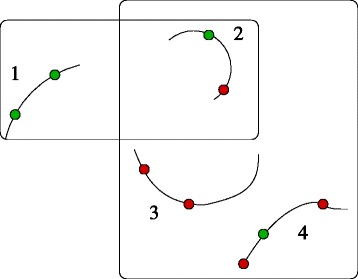
Four nets involved in two tests. The points of interest pictured in green are those that can be touched by at least one probe. Those pictured in red cannot be touched by any probe. The test consisting of nets 1 and 2 can be carried out in the current configuration but not the test consisting of nets 2, 3, and 4

Given a subset *R* of nets, a *test* consists of assigning certain probes to nets in *R* so that one point in each net is touched by one probe; the set of probes that may be used to touch a given point is known in advance and depends upon the test being carried out. Hence a test consists, formally, of a collection of nets and a family of probe sets (one set of probes for each point in each net involved in the test). The probes must touch the points simultaneously, which means that there must be a matching between a subset *Q* of the probes and the collection *R* of nets. Of course such a matching need not exist: its existence depends upon the locations of the eight shuttles. Figure 1 displays two tests, one of which can be carried out in the current configuration but the other cannot.

In general, given a *configuration of shuttles*, only a fraction of the tests can be carried out. Thus several configurations must be computed in order to ensure that all the tests are covered. (Note that the initial configuration, which is also the final one, is given in advance.) Moving the shuttles from one configuration to the next requires some time, which can be evaluated if one knows the coordinates of the shuttles within their successive configurations. The objective of the Company is to minimize the total time needed to move the shuttles between configurations, with the constraint that each test is covered by at least one configuration. The system currently used by the Company yields poor results, in the sense that the number of configurations needed to cover all the tests is far too large, resulting in many unnecessary shuttle moves. Hence the problem was proposed to us by the Company at the Fields-MITACS Industrial Problem-Solving Workshop (see Chen S, Gustafsson J, Marcotte O, Morfin M, Pugh M: Improved optimization for a testing system with two mobility layers, in Proceedings of the Fields-MITACS Industrial Problem-Solving Workshop, forthcoming).

The *planning problem* just described is similar to the Travelling Salesman Problem (TSP), one of the basic problems in combinatorial optimization (see Applegate et al. [2]). We conjecture that in its general form, the planning problem is as difficult to solve as the TSP. Although we are not aware of any previous work on the planning problem, there are similarities between our problem and two classes of problems that have been considered already: geometric covering problems (see for instance the article by Arkin and Hassin [3] and the article by Ahn et al. [1]) and some generalizations of the TSP. One of these generalizations is the Travelling Politician Problem (see Riordan [19]). Other generalizations include the Covering Salesman Problem (see the article by Current and Schilling [5]) and the Clustered Travelling Salesman Problem (CTSP), discussed in an article by Laporte and Palekar [11] that includes an application of the CTSP to a circuit design problem.

The Generalized Travelling Salesman Problem (GTSP) has received a fair amount of attention (see for example the articles [12–15] by Laporte and his colleagues). Some authors have proposed transforming a GTSP instance into a TSP instance (for an example see Dimitrijevic and Saric [6]). Others have proposed a Lagrangian-based approach for the asymmetric GTSP (Noon and Bean [17]) or a branch-and-cut algorithm for the symmetric GTSP (Fischetti et al. [8]). Fischetti et al. [7] have studied the symmetric GTSP polytope. Recently Pop [18] and Kara, Guden, and Koc [10] have proposed new formulations for the GTSP. In spite of the similarities between the planning problem and the various problems we have just mentioned, our problem seems to be completely new and a major portion of the present article will be devoted to its formulation as a mathematical program.

As mentioned before, although the objective of the Company is to minimize the total time needed to move the shuttles between configurations, the main difficulty faced by the Company is the large number of configurations required to cover all the tests in the system that it currently uses. Hence our first objective is to try to reduce the number of configurations. To achieve this we tackle the planning problem in two steps. First we construct a partition of the set of tests into subsets that can be carried out within a single configuration. We call this problem the *covering problem* since we are looking for a family of configurations that covers each and every test. Then we solve the *optimal sequence problem* in order to find the best sequence of configurations (using only those configurations that are in the solution of the covering problem). Since the decomposition of the planning problem into a covering problem and a sequencing problem does not always yield an optimal solution, we also present a global formulation of the planning problem.

Our article is organized as follows. The “Methods” section deals with the modelling and solution of the planning problem. It includes a subsection on shuttles and probes; a subsection on power chains and avoidance of collisions; a subsection on probes, points of interest, and reachability constraints; subsections on incompatibility constraints and tests and feasibility constraints, respectively; a subsection on the partial covering problem; a subsection on a greedy algorithm for the covering problem; a subsection on the optimal sequence problem; and finally a subsection on a global formulation of the planning problem. The article continues with a section on “Results and discussion” and ends with the “Conclusion” section.

## Methods

**Shuttles and probes**

We assign the indices 0,1,…,7 to the eight *shuttles* in the following way: the shuttles that are above the board are labelled 0, 1, 2, 3 in the clockwise order starting with the front-left shuttle and the shuttles below are labelled 4, 5, 6, 7 in a similar way. Hence Shuttle 0 is the front-left shuttle that is above the board, Shuttle 1 is the back-left shuttle above the board, Shuttle 2 the back-right shuttle above the board, and so on. The dimensions of each shuttle are 195 mm (in the horizontal direction) and 160 mm (in the vertical direction). The dimensions of the board are 1050 mm (in the horizontal direction) and 850 mm (in the vertical direction). We will use these constants in the model but obviously they can be replaced by other constants if the covering problem must be solved for another system. Each shuttle has a *preferred corner*, corresponding to the point where its *power chain* is attached to the board. For instance, the preferred corner of the front-left shuttle that is above (resp. below) the board is the front-left corner of this shuttle.

The *initial configuration* is the configuration in which the preferred corner of each shuttle is located at the corresponding board corner. For instance, the initial coordinates of the preferred corner of the back-left shuttle are (0,850). A given board cannot be introduced into (or removed from) the FP system unless it is in the initial configuration. Hence when the board is introduced into the FP system, one computes the tests that can be carried out within this configuration before considering other configurations.

In our model the *x*-coordinate of Shuttle *k* (for *k*∈{0,1,…,7}) will be denoted by *S*_2*k*_ and its *y*-coordinate by *S*_2*k*+1_: those two expressions are variables since we are looking for “good” configurations and the goal of the model is to find them. The *probes*, which can come into contact with *points of interest* (or points, for short), are attached to their respective shuttles. The number of probes in the system under consideration is 21. The expression *k*(*p*) will denote the shuttle to which the probe *p* is attached, for *p* in {0,1,2,…,20}. For each shuttle we also know its *x*-offset and *y*-offset, denoted respectively by *O*_*p*1_ and *O*_*p*2_. (Note that *O*_*p*1_ and *O*_*p*2_ are constants, not variables.) The coordinates of the point where the probe *p* is attached to the shuttle *k*(*p*) are thus equal to *S*_2*k*(*p*)_+*O*_*p*1_ and *S*_2*k*(*p*)+1_+*O*_*p*2_, respectively.

**Power chains and avoidance of collisions**

In this subsection we first address the problem of modelling the avoidance of collisions above the board, specifically collisions

- between the power chain of a given shuttle and any other shuttle;
- between the power chains of any two shuttles; and
- between two shuttles.

**Fig. 2:**
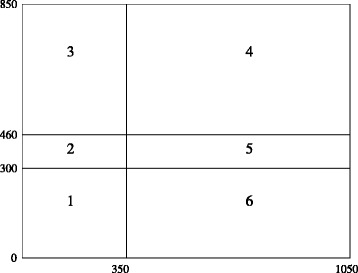
The six regions considered when studying the collisions between the front-left shuttle and another shuttle

**Fig. 3:**
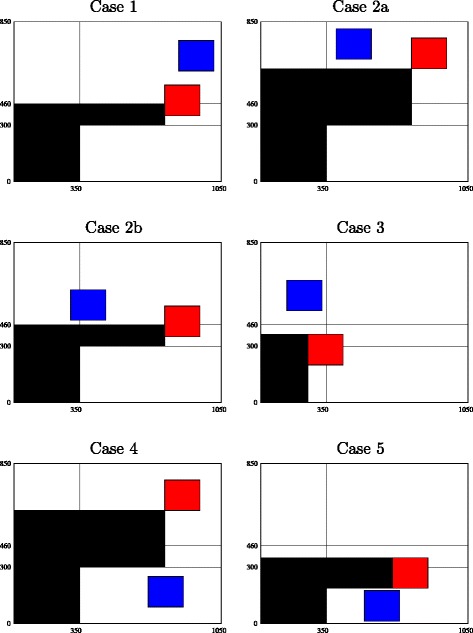
Illustration of the various cases when studying the collisions between the front-left shuttle and another shuttle : in Case 1 the other shuttle is included within the vertical strip, in Cases 2a, 2b, and 3 within the upper horizontal strip, and in Cases 4 and 5 within the lower horizontal strip (see the text for more details)

Figure 2 describes the board regions considered when studying collisions. We will first consider modelling the avoidance of collisions between the power chain of a given shuttle and any other shuttle. Figure 3 illustrates the *forbidden region* corresponding to the front-left shuttle for each of five cases (note that Case 2 is illustrated by two subcases), where the forbidden region denotes (roughly) the region that can be occupied by the power chain of the front-left shuttle. Each of the five cases is described precisely below. Our definition of forbidden region is too strict, in the sense that a shuttle or power chain could occupy part of the forbidden region without interfering with the power chain of the shuttle under consideration. A more precise definition, however, would be very difficult to model, since a power chain is a flexible object containing several springs and may assume many different positions.

We now give the (mathematical) description of forbidden region that we will use. Let (*S*_0_,*S*_1_) denote the coordinates of the preferred corner of the front-left shuttle. We first define the *large rectangle*: if *S*_1_ is at least 460, the large rectangle denotes the set

$$\{(x,y) \> | \> 0 \leq x \leq S_{0}, 0 \leq y \leq S_{1} \}; $$

if *S*_1_ is less than 460, it denotes the set

$$\{(x,y) \> | \> 0 \leq x \leq S_{0}, 0 \leq y \leq \text{min}(S_{1}+160,460) \}. $$

If *S*_0_ is at most 350, the forbidden region is precisely the large rectangle. If *S*_0_ is greater than 350, the forbidden region is the set difference between the large rectangle and the *small rectangle*, where the latter is defined as

$$\{(x,y) \> | \> 350 \leq x \leq S_{0}, 0 \leq y \leq \text{min}(S_{1},300) \}. $$

We now turn to the five cases that arise when considering the avoidance of collisions between the power chain of the front-left shuttle and any other shuttle. In Fig. 3 the front-left shuttle is depicted in red, the other shuttle in blue, and the forbidden region in black.

Let $S^{\prime }_{0}$ and $S^{\prime }_{1}$ denote the coordinates of the *avoidance corner* of the “other” shuttle (which is anonymous for the time being). This avoidance corner is the front-left corner of the other shuttle. Here are the five cases, which are not mutually exclusive:

1. the other shuttle is included within the vertical strip (i.e., $S^{\prime }_{0} \geq S_{0}$ holds);
2. the other shuttle is included within the upper horizontal strip (i.e., $S^{\prime }_{1} \geq \text {max} \{460,S_{1}\}$ holds) and *S*_1_ is at least equal to 300;
3. the other shuttle is included within the upper horizontal strip (i.e., $S^{\prime }_{1} \geq S_{1} + 160$ holds) and *S*_1_ is at most equal to 300;
4. the other shuttle is included within the lower horizontal strip (i.e., $S^{\prime }_{0} \geq 350$ and $S^{\prime }_{1} + 160 \leq 300$ hold), *S*_0_ is at least equal to 350, and *S*_1_ at least equal to 300;
5. the other shuttle is included within the lower horizontal strip (i.e., $S^{\prime }_{0} \geq 350$ and $S^{\prime }_{1} + 160 \leq S_{1}$ hold), *S*_0_ is at least equal to 350, and *S*_1_ at most equal to 300.

To model these collision avoidance constraints, we define five binary variables *y*_*r*_ (for *r*∈{0,1,2,3,4}), each of which corresponding to one of the cases listed above. The first constraint below means that one of the five cases must be chosen in order to verify that the avoidance corner (i.e., the front-left corner of the “other” shuttle) does not belong to the forbidden region. The second, third, and fourth constraints ensure that if *y*_*i*−1_ (for *i* in {1,2,3,4,5}) has the value 1, the conditions on *S*_0_ and *S*_1_ in Case *i* are indeed verified. The fifth (resp. eighth) constraint ensures that the coordinates $S^{\prime }_{0}$ and $S^{\prime }_{1}$ satisfy the conditions in Case 1 (resp. 3). Finally the sixth and seventh (resp. ninth and tenth, eleventh and twelfth) constraints ensure that $S^{\prime }_{0}$ and $S^{\prime }_{1}$ satisfy the conditions in Case 2 (resp. 4, 5).

$$\begin{array}{*{20}l} y_{0} + y_{1} + y_{2} + y_{3} + y_{4} & = 1 \\ S_{0} & \geq 350 (y_{3} + y_{4}) \\ S_{1} & \geq 300 (y_{1} + y_{3}) \\ S_{1} & \leq 300 (y_{2} + y_{4}) + 850 (1 - y_{2} - y_{4}) \\ S'_{0} - S_{0} & \geq -1050 (1 - y_{0}) \\ S'_{1} - 460 & \geq -850 (1 - y_{1}) \\ S'_{1} - S_{1} & \geq -850 (1 - y_{1}) \\ S'_{1} - S_{1} - 160 & \geq -850 (1 - y_{2}) \\ S'_{0} & \geq 350 y_{3} \\ S'_{1} + 160 & \leq 300 y_{3} + 850 (1 - y_{3}) \\ S'_{0} & \geq 350 y_{4} \\ S'_{1} + 160 - S_{1} & \leq 850 (1 - y_{4}) \end{array} $$

We now simplify these constraints as follows.

1 $$ y_{0} + y_{1} + y_{2} + y_{3} + y_{4} = 1  $$

2 $${} -S_{0} + 350 y_{3} + 350 y_{4} \leq 0  $$

3 $${} -S_{1} + 300 y_{1} + 300 y_{3} \leq 0 \\  $$

4 $$ S_{1} + 550 y_{2} + 550 y_{4} \leq 850  $$

5 $$ -S'_{0} + S_{0} + 1050 y_{0} \leq 1050  $$

6 $${\kern23pt} -S'_{1} + 850 y_{1} \leq 390  $$

7 $${\kern2pt} -S'_{1} + S_{1} + 850 y_{1} \leq 850  $$

8 $${\kern2pt} -S'_{1} + S_{1} + 850 y_{2} \leq 690  $$

9 $${\kern23pt} -S'_{0} + 350 y_{3} \leq 0  $$

10 $${\kern35pt} S'_{1} + 550 y_{3} \leq 690  $$

11 $${\kern23pt} -S'_{0} + 350 y_{4} \leq 0  $$

12 $${\kern14pt} S'_{1} - S_{1} + 850 y_{4} \leq 690  $$

The above constraints are “abstract” in the sense that $S^{\prime }_{0}$ and $S^{\prime }_{1}$ represent the avoidance corner of an anonymous shuttle. Actually, to model the requirement that there is no collision between the power chain of the front-left shuttle and any other shuttle, we need three groups of 12 constraints, each corresponding to one of the following shuttles: the back-left shuttle ($S^{\prime }_{0} = S_{2}$, $S^{\prime }_{1} = S_{3} - 160$), the back-right shuttle ($S^{\prime }_{0} = S_{4} - 195$, $S^{\prime }_{1} = S_{5} - 160$), and the front-right shuttle ($S^{\prime }_{0} = S_{6} - 195$, $S^{\prime }_{1} = S_{7}$). We then obtain a complete set of constraints for the front-left shuttle, i.e., 36 constraints. This set requires the introduction of 15 binary variables. We need similar sets of constraints and binary variables for the other shuttles. In total there will be 60 binary variables and 144 constraints.

We must now model the requirement that two avoidance regions cannot overlap, so as to ensure that two power chains cannot “cross.” Consider the avoidance region of the front-left shuttle and its relationships with the other avoidance regions.

- Given that no two shuttles intersect (see below) and no shuttle has a non-empty intersection with the avoidance region of any other shuttle, it is enough to impose the condition *S*_3_≥*S*_1_ in order to prevent the avoidance regions of the front-left and back-left shuttles from having a non-empty intersection.
- The back-right shuttle cannot belong to the lower horizontal region (or at least the part of the lower horizontal region that is not already in the vertical region). Otherwise the avoidance region of the back-right shuttle would have a non-empty intersection with the front-left shuttle **or** the avoidance region of the front-left shuttle. In practice this means that the variables *y*_8_ and *y*_9_ must equal 0.
- The front-right shuttle cannot belong to the upper horizontal region (or at least the part of the upper horizontal region that is not already in the vertical region). Otherwise the avoidance region of the front-right shuttle would have a non-empty intersection with the front-left shuttle **or** the avoidance region of the front-left shuttle. In practice this means that the variables *y*_11_ and *y*_12_ must equal 0.

The above conditions will ensure that there is no collision between the power chains in pairs of shuttles that include the front-left shuttle. Similar conditions are needed for the three other pairs of shuttles. Therefore to avoid collisions between power chains (on one hand) and shuttles or other power chains (on the other), we can use the above constraints (after forcing some variables to equal 0) and add the following constraints to the model.

13 $$\begin{array}{*{20}l} S_{3} \geq S_{1} \end{array} $$

14 $$\begin{array}{*{20}l} S_{5} \geq S_{7} \end{array} $$

Finally we turn to collisions between shuttles. We consider again the front-left shuttle and assume that $S^{\prime }_{0}$ and $S^{\prime }_{1}$ are the coordinates of the front-left corner of the “other” shuttle. The variable *y*_60_ (resp. *y*_61_, *y*_62_, *y*_63_) takes the value 1 if and only if the “other” shuttle is to the left (resp. above, to the right, below) of the front-left shuttle. The following constraints ensure that the two shuttles will not overlap.

$$\begin{array}{*{20}l} y_{60} + y_{61} + y_{62} + y_{63} & = 1 \\ S'_{0} + 195 & \leq S_{0} + 1050 (1 - y_{60}) \\ S'_{1} + 850 (1 - y_{61}) & \geq S_{1} + 160 \\ S'_{0} + 1050 (1 - y_{62}) & \geq S_{0} + 195 \\ S'_{1} + 160 & \leq S_{1} + 850 (1 - y_{63}) \end{array} $$

We obtain the following system after simplifying them.

15 $$\begin{array}{*{20}l} y_{60} + y_{61} + y_{62} + y_{63} & = 1 \end{array} $$

16 $$\begin{array}{*{20}l} - S_{0} + S'_{0} + 1050 y_{60} & \leq 855 \end{array} $$

17 $$\begin{array}{*{20}l} S_{1} - S'_{1} + 850 y_{61} & \leq 690 \end{array} $$

18 $$\begin{array}{*{20}l} S_{0} - S'_{0} + 1050 y_{62} & \leq 855 \end{array} $$

19 $$\begin{array}{*{20}l} - S_{1} + S'_{1} + 850 y_{63} & \leq 690 \end{array} $$

As before, the variables $S^{\prime }_{0}$ and $S^{\prime }_{1}$ must be replaced by the correct expressions for every shuttle other than the front-left shuttle. The three other pairs (back-left and back-right, back-left and front-right, back-right and front-right) must also be taken into account. For this kind of constraints a total of 24 binary variables and 30 relations are needed.

We now have a complete system of constraints preventing collisions between shuttles and power chains that are above the board. Of course a similar system is required for the shuttles and power chains that are below the board. All these constraints will be referred to as the *collision avoidance constraints* and denoted by *CAC*(*S*_*u*_,*y*_*r*_), where the variables of the form *y*_*r*_ are the *auxiliary variables*.

**Probes, points of interest, and reachability constraints**

We now introduce the binary variables *X*_*αp*_ for every point *α* and every probe *p*, where *X*_*αp*_ is a binary variable that equals 1 if and only if probe *p* (attached to the shuttle *k*(*p*)) can reach the point *α* (whose coordinates are denoted by *P*_*α*1_ and *P*_*α*2_, respectively). We wish to model the constraint that if *X*_*αp*_ equals 1, then the probe *p* can actually reach the point *α*. Given the system we are studying, this amounts to saying that if *X*_*αp*_ equals 1, the expression |*S*_2*k*(*p*)_+*O*_*p*1_−*P*_*α*1_| should be at most 30.0 and the expression |*S*_2*k*(*p*)+1_+*O*_*p*2_−*P*_*α*2_| at most 33.5. In other words, if *X*_*αp*_ equals 1, the following relations must be satisfied.

$$\begin{array}{*{20}l} S_{2k(p)} + O_{p1} - P_{\alpha 1} & \leq 30.0 \\ -S_{2k(p)} - O_{p1} + P_{\alpha 1} & \leq 30.0 \\ S_{2k(p)+1} + O_{p2} - P_{\alpha 2} & \leq 33.5 \\ -S_{2k(p)+1} - O_{p2} + P_{\alpha 2} & \leq 33.5 \end{array} $$

We can rewrite them as follows.

$$\begin{array}{*{20}l} S_{2k(p)} & \leq -O_{p1} + P_{\alpha 1} + 30.0 \\ -S_{2k(p)} & \leq O_{p1} - P_{\alpha 1} + 30.0 \\ S_{2k(p)+1} & \leq - O_{p2} + P_{\alpha 2} + 33.5 \\ -S_{2k(p)+1} & \leq O_{p2} - P_{\alpha 2} + 33.5 \end{array} $$

The variable *S*_2*k*(*p*)_ (resp. *S*_2*k*(*p*)+1_) must satisfy the above constraints if *X*_*αp*_ equals 1; otherwise it is bounded from above (resp. below) by its upper (resp. lower) bound. Here are the constraints that we include into the model.

20 $$\begin{array}{*{20}l} S_{2k(p)} & \leq \left(-O_{p1} + P_{\alpha 1} + 30.0 \right) X_{\alpha p} + UB_{2k(p)} \left(1.0 - X_{\alpha p} \right) \end{array} $$

21 $$\begin{array}{*{20}l} -S_{2k(p)} & \leq \left(O_{p1} - P_{\alpha 1} + 30.0 \right) X_{\alpha p} - LB_{2k(p)} \left(1.0 - X_{\alpha p} \right) \end{array} $$

22 $$\begin{array}{*{20}l} S_{2k(p)+1} & \leq \left(-O_{p2} + P_{\alpha 2} + 33.5 \right) X_{\alpha p}+ UB_{2k(p)+1} \left(1.0 - X_{\alpha p} \right) \end{array} $$

23 $$\begin{array}{*{20}l} -S_{2k(p)+1} & \leq \left(O_{p2} - P_{\alpha 2} + 33.5 \right) X_{\alpha p} - LB_{2k(p)+1} \left(1.0 - X_{\alpha p} \right) \end{array} $$

These inequalities (called *reachability constraints*) are required for each couple (*α*,*p*). The set of reachability constraints will be denoted by *REACH*(*S*_*u*_,*X*_*αp*_).

**Incompatibility constraints**

These constraints are not necessary for modelling our problem but allow us to strengthen its linear programming relaxation. Assume that *α* and *β* denote two points of interest and *p* and *q* two probes attached to the same shuttle. It is easy to derive from the reachability constraints some conditions implying that *X*_*αp*_ and *X*_*βq*_ cannot both equal 1. So for each pair of couples (*α*,*p*) and (*β*,*q*) satisfying those conditions, we may add the constraint *X*_*αp*_+*X*_*βq*_≤1 to the model. In practice we do not add all the incompatibility constraints to the model: rather we solve its linear relaxation and add to the model all such constraints that are violated in the current optimal solution and verify *X*_*αp*_≥0.2 and *X*_*βq*_≥0.2 in the current optimal solution. The set of all incompatibility constraints included into the model will be denoted by *INC*(*X*_*αp*_).

**Tests and feasibility constraints**

**Fig. 4:**
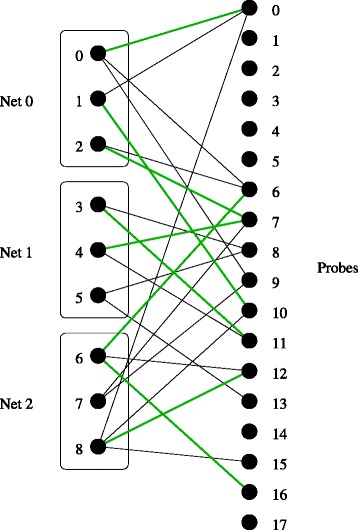
The graph for a specific test (assuming the number of probes equals 18): the set of all the edges represented is *E*_*ℓ*_ while the green edges are those defining the graph *G*_*ℓ*_

Given the current configuration (i.e., a set of values for the variables *S*_*u*_ representing the shuttles coordinates), we wish to determine whether a specific test can be carried out or not. We introduce, for each *ℓ*, a binary variable *Y*_*ℓ*_ that may take the value 1 only if the test *ℓ* can be carried out within the current configuration. We must find a way of relating the value of *Y*_*ℓ*_ to those of the *X*_*αp*_. First we show that the test *ℓ* can be performed within the current configuration if and only if there is a matching of a certain type in a bipartite graph (denoted *G*_*ℓ*_) that we now describe. Let *N*_*ℓ*_ denote the set of nets involved in test *ℓ* and *U*_*ℓ*_ the set of points belonging to some net in *N*_*ℓ*_. The graph *G*_*ℓ*_ contains two kinds of vertices: the points in *U*_*ℓ*_ and the probes. It contains an edge between point *α* and probe *p* if and only if probe *p* can be used to touch point *α* within the test *ℓ* (i.e., the couple (*α*,*p*) is *admissible*) and *X*_*αp*_ equals 1. Recall that the admissibility of the couple (*α*,*p*) depends upon *ℓ*. We denote by *E*_*ℓ*_ the set of admissible couples for test *ℓ*. In Fig. 4 all the edges in *E*_*ℓ*_ are represented: the green edges are those defining graph *G*_*ℓ*_ and the black edges represent couples (*α*,*p*) that are admissible but verify *X*_*αp*_=0. Note that Net 0 (resp. Net 1, Net 2) is the set {0,1,2} (resp. {3,4,5}, {6,7,8}).

From the definition of “carrying out a test,” it follows that a test can be performed within the current configuration if and only if *G*_*ℓ*_ contains a matching saturating exactly one point within each net in *N*_*ℓ*_. For instance the green graph *G*_*ℓ*_ in Fig. 4 contains the matching {(1,10),(4,7),(8,12)}, showing that probe 10 (resp. 7, 12) can touch point 1 (resp. 4, 8) in Net 0 (resp. 1, 2). Hence the test *ℓ* can be carried out within the current configuration. We now observe that finding a matching saturating exactly one point within each net in *N*_*ℓ*_ is equivalent to finding a system of distinct representatives for the collection of sets {*Z*_*i*_}, where *Z*_*i*_ is the set of probes that can be used to touch a point in the net *i*, i.e.,

$$Z_{i} = \left \{p \in P \> | \> (\alpha,p) \text{~is admissible and~} X_{\alpha p} = 1 \text{~for some~} \alpha \text{~in net~} i \right \}$$

and *P* denotes the set of probes. A classical theorem in combinatorial optimization asserts that the collection of sets $\phantom {\dot {i}\!}\{Z_{i}\}_{i \in N_{\ell }}$ has a system of distinct representatives if and only if the condition

$$\left | \bigcup_{i \in M} Z_{i} \right | \geq |M| $$

is satisfied for every subset *M* of *N*_*ℓ*_ (see in particular the seminal text by Ford and Fulkerson [9] and the book by Chvátal [4]). This theorem can be rephrased as follows: the collection $\phantom {\dot {i}\!}\{Z_{i}\}_{i \in N_{\ell }}$ has a system of distinct representatives if and only if the condition

$$\left(\bigcup_{i \in M} Z_{i} \right) \cap Q \neq \emptyset $$

holds for every set *Q* verifying $|\overline {Q}| = |M|-1$ (where $\overline {Q}$ denotes the complement of *Q*).

Let *M* denote any nonempty subset of *N*_*ℓ*_ and *U*(*M*) the union of all the points belonging to nets in *M*. The statement

$$\left(\bigcup_{i \in M} Z_{i} \right) \cap Q \neq \emptyset $$

is equivalent to the statement

$$\sum_{\underset{\alpha \in U(M),p \in Q}{(\alpha,p) \in E_{\ell}}} X_{\alpha p} \geq 1,$$

by the definition of the *Z*_*i*_. We thus obtain the following proposition.

### **Proposition 2.1**

The graph *G*_*ℓ*_ contains a matching saturating exactly one point within each net in *N*_*ℓ*_ if and only if the inequalities

$$\sum_{\underset{\alpha \in U(M),p \in Q}{(\alpha,p) \in E_{\ell}}} X_{\alpha p} \geq 1$$

hold for all sets *M*, *Q* such that *M* is not empty, *Q* is a subset of probes, and $|\overline {Q}|$ equals |*M*|−1 (where $\overline {Q}$ denotes the complement of *Q*).

For each test *ℓ* we include into our model the constraints

24 $$\begin{array}{*{20}l} \sum_{\underset{\alpha \in U(M),p \in Q}{(\alpha,p) \in E_{\ell}}} X_{\alpha p} \geq Y_{\ell} \end{array} $$

for all sets *M*, *Q* such that *M* is not empty, *Q* is a subset of probes, and $|\overline {Q}|$ equals |*M*|−1. They will be called *feasibility constraints* and denoted by *FEAS*(*X*_*αp*_,*Y*_*ℓ*_). Proposition 2.1 implies that if *Y*_*ℓ*_ equals 1, then the graph *G*_*ℓ*_ contains a matching of the required type and test *ℓ* can be carried out within the current configuration.

For an illustration we give the feasibility constraints for the test represented in Fig. 4. First we assume that |*M*| equals 1 and write the following constraints.

$$\begin{array}{*{20}l} X_{0,0} + X_{0,6} + X_{0,9} + X_{1,0} + X_{1,10} + X_{2,6} + X_{2,7} \geq Y_{\ell} \\ X_{3,8} + X_{3,11} + X_{4,7} + X_{4,11} + X_{5,8} + X_{5,13} \geq Y_{\ell} \\ X_{6,6} + X_{6,12} + X_{6,16} + X_{7,7} + X_{7,9} + X_{8,0} + X_{8,10} + X_{8,12} + X_{8,15} \geq Y_{\ell} \end{array} $$

For the case where |*M*| equals 2, we need all the constraints of the form

$$\begin{array}{*{20}l} \sum_{\underset{\alpha \in U(M),p \neq q}{(\alpha,p) \in E_{\ell}}} X_{\alpha p} \geq Y_{\ell} \end{array} $$

for some probe *q*. For instance, if *M* consists of nets 0 and 1 and *q* equals 6, we have

$$\begin{array}{*{20}l} X_{0,0} + X_{0,9} + X_{1,0} + X_{1,10} + X_{2,7} + X_{3,8} + X_{3,11} + X_{4,7} + X_{4,11} + X_{5,8} + X_{5,13} \geq Y_{\ell}. \end{array} $$

Finally, for the case where |*M*| equals 3, we need all the constraints of the form

$$\begin{array}{*{20}l} \sum_{\underset{\alpha \in U(M), p \neq q,q'}{(\alpha,p) \in E_{\ell}}} X_{\alpha p} \geq Y_{\ell} \end{array} $$

for some pair of probes {*q,q* ^′^}. For instance, if *q* equals 6 and *q* ^′^ equals 7, we have

$${} \begin{aligned} X_{0,0} &+ X_{0,9} + X_{1,0} + X_{1,10} + X_{3,8} + X_{3,11} + X_{4,11} + X_{5,8} + X_{5,13} + X_{6,12} + X_{6,16} + X_{7,9}\\ &+ X_{8,0} + X_{8,10} + X_{8,12} + X_{8,15} \geq Y_{\ell}. \end{aligned} $$

When the model containing these feasibility constraints returns a solution, one must do a little bit of work to compute the couples (*α*,*p*) that describe the implementation of test *ℓ* (for each *ℓ*). Actually it suffices to solve a bipartite matching problem in the graph *G*_*ℓ*_ corresponding to this solution. We now comment on the number of feasibility constraints for a given *ℓ*. Assuming that there are 21 probes (as in the case of our data sets), this number equals

$$\sum_{m=1}^{|N_{\ell}|} \binom{|N_{\ell})|}{m} \sum_{Q:|Q| = 21-(m-1)} 1 = \sum_{m=1}^{|N_{\ell}|} \binom{|N_{\ell}|}{m} \binom{21}{m-1} $$

and grows rapidly as a function of |*N*_*ℓ*_|. This is not an issue because in our data sets |*N*_*ℓ*_| is almost always less than five. Nonetheless one might ask why we do not model directly the requirement that there be a matching between nets and probes (in order to carry out a specific test). The reason is that to do so, we would need to introduce variables of the form *X*_*αpℓ*_ and the resulting model would contain too many variables.

If one does not want to use the above feasibility constraints when |*N*_*ℓ*_| is at least three (note that the number of constraints equals 276 when *N*_*ℓ*_ contains three nets!), an alternative is to introduce the supplementary variables *X*_*αpℓ*_ and the following constraints.

$$\begin{array}{*{20}l} \sum_{\underset{\alpha \in U \left(N_{\ell} \right)} {(\alpha,p) \in E_{\ell}}} X_{\alpha p \ell} & \leq 1 \text{~for all~} p \in P \\ Y_{\ell} \leq \sum_{\underset{\alpha \in k, p \in P}{(\alpha,p) \in E_{\ell}}} X_{\alpha p \ell} & \leq 1 \text{~for all~} k \in N_{\ell} \\ X_{\alpha p \ell} & \leq X_{\alpha p} \text{~for all~} (\alpha, p, \ell) \end{array} $$

**The partial covering problem**

It is natural to ask the following question: what is the maximum number of tests that can be carried out while the system is in a given configuration? This problem, which we call the *partial covering problem*, can be formulated as follows: maximize the function $\sum _{\ell } Y_{\ell }$ subject to the collision avoidance constraints *CAC*(*S*_*u*_,*y*_*r*_), the reachability constraints *REACH*(*S*_*u*_,*X*_*αp*_), the incompatibility constraints *INC*(*X*_*αp*_), the feasibility constraints *FEAS*(*X*_*αp*_,*Y*_*ℓ*_), the lower and upper bound constraints on all the variables, and the integrality constraints on the binary variables. The resulting mixed integer linear program can be solved by using a commercial solver.

**A greedy algorithm for the covering problem**

The covering problem can be solved in a heuristic fashion through repeated calls of an algorithm for solving the partial covering problem. This method is summarized in Algorithm 1. Note that in the first line of the loop body, ${\mathcal {T}}$ can be replaced by a subset of tests. Then one must determine which of the omitted tests are covered by the configuration *C* before removing them from ${\mathcal {T}}$.


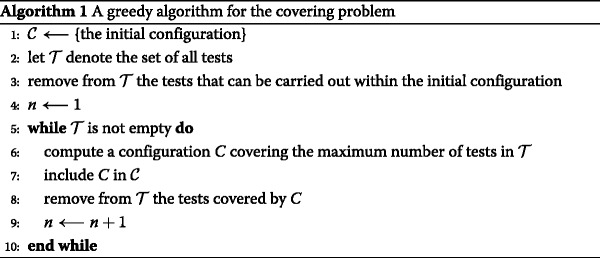


**The optimal sequence problem**

Let ${\mathcal {C}}$ be the set of configurations computed by the above algorithm and *n* its cardinality. Thus ${\mathcal {C}}$ is of the form {*C*_1_,*C*_2_,…,*C*_*n*_}, where *C*_*i*_ denotes the *i*th configuration (*C*_1_ being the initial configuration). Let also *d*(*C*_*i*_,*C*_*j*_) denote the time taken by the FP system to go from the configuration *C*_*i*_ to the configuration *C*_*j*_. We wish to compute the best sequence of configurations, i.e., a sequence (*C*_1_=*C*_*σ*(1)_,*C*_*σ*(2)_,…,*C*_*σ*(*n*)_,*C*_1_) (where *σ* denotes a permutation) that minimizes

$$\sum \{d \left(C_{i},C_{j} \right) \> | \> j \text{~follows~} i \text{~in the sequence}\}.$$

When a shuttle moves from one configuration to the next, it travels at the same speed in the horizontal direction as in the vertical direction, and thus the time it takes to do so is proportional to the *L*_*∞*_ distance between the respective positions of the shuttle in the two configurations. Hence we assume that *d*(*C*_*i*_,*C*_*j*_) is the *L*_*∞*_ distance between *C*_*i*_ and *C*_*j*_.

Since the distances are symmetric (meaning that *d*(*C*_*i*_,*C*_*j*_)=*d*(*C*_*j*_,*C*_*i*_) holds for any pair {*i,j*}), we can model the optimal sequence problem as a Travelling Salesman Problem (TSP) on an undirected graph. The TSP itself can be modelled by using *degree constraints* (each vertex must be of degree 2) and *subtour elimination constraints* (enforcing the condition that there be at least two edges between a subset *S* of vertices and its complement, for any *S* verifying 2≤|*S*|≤*n*−2). We introduce the binary variables *z*_*ij*_ for 1≤*i*<*j*≤*n*, where *z*_*ij*_ equals 1 if and only if the edge *ij* belongs to the tour. Here is the integer programming formulation of the optimal sequence problem.

$$\min \quad \sum\limits_{i=1}^{n-1} \sum\limits_{j=i+1}^{n} d \left(C_{i},C_{j} \right) z_{ij} $$

subject to

25 $$\begin{array}{@{}rcl@{}} \sum\limits_{j=1}^{i-1} z_{ji} + \sum\limits_{j=i+1}^{n} z_{ij} = 2 && \quad \forall i \in \{1,2,\dots,n\}  \end{array} $$

26 $$\begin{array}{@{}rcl@{}} \sum\limits_{i \in S} \sum\limits_{\underset{j>i}{j \notin S}} z_{ij} + \sum\limits_{i \in S} \sum\limits_{\underset{j<i}{j \notin S}} z_{ji} \geq 2 && \quad \forall S \subset \{1,2,\dots,n\}, 2 \leq |S| \leq n-2  \\ z_{ij} \in \{0,1\} && \quad \forall i, \forall j \text{~such that~} i<j  \end{array} $$

**A global formulation of the planning problem**

We now give a formulation of the planning problem that is global but unlikely to be solved for large data sets. In this formulation we are looking for several configurations at once, so that each test is covered by at least one configuration. We assume that there are at most *n* configurations. We introduce a group of variables $\left (S^{i}_{u}, y^{i}_{r}, X^{i}_{\alpha p}, Y^{i}_{\ell } \right)$ for each *i*∈{1,2,…,*n*}, that is, for each potential configuration. The variable $Y^{i}_{\ell }$ equals 1 if and only if the test *ℓ* is covered by the configuration represented by the shuttles coordinates $S^{i}_{u}$. For each *i* the vector $\left (S^{i}_{u}, y^{i}_{r}, X^{i}_{\alpha p}, Y^{i}_{\ell } \right)$ must satisfy the constraints in $CAC \left (S^{i}_{u}, y^{i}_{r} \right)$, $REACH \left (S^{i}_{u}, X^{i}_{\alpha p} \right)$, $INC \left (X^{i}_{\alpha p} \right)$, and $FEAS \left (X^{i}_{\alpha p}, Y^{i}_{\ell } \right)$, as well as the bound and nonnegativity constraints on the variables and the integrality constraints on all the variables except the $S^{i}_{u}$.

To these constraints one must add constraints ensuring that each test is covered by at least one configuration. These constraints can be expressed as $\sum _{i=1}^{n} Y^{i}_{\ell } \geq 1$ for every *ℓ*. We now introduce variables and constraints to model the choice of the optimal configuration sequence. For *i* in {1,2,…,*n*}, let *w*_*i*_ be a binary variable that equals 1 if and only if Configuration *i* is chosen. For any nonempty proper subset *S* of {1,2,…,*n*}, let *w*_*S*_ be a binary variable that equals 1 if and only if *S* contains at least one of the chosen configurations. As in the subsection “Methods”, we introduce the variables *z*_*ij*_ defined as follows: *z*_*ij*_ (for 1≤*i*<*j*≤*n*) equals 1 if and only if Configuration *i* appears immediately before or after Configuration *j* in the sequence of configurations. Finally we let *d*_*ij*_ denote the *L*_*∞*_ distance between Configurations *i* and *j*. We can now propose a model for choosing an optimal configuration sequence.

$$\min \quad \sum\limits_{i=1}^{n-1} \sum\limits_{j=i+1}^{n} d_{ij} z_{ij} $$

subject to

27 $$\begin{array}{@{}rcl@{}} \sum\limits_{i=1}^{n} Y^{i}_{\ell} \geq 1 && \quad \forall \ell \end{array} $$

28 $$\begin{array}{@{}rcl@{}} Y^{i}_{\ell} \leq w_{i} && \quad \forall i \in \{1,2,\dots,n\}, \forall \ell \end{array} $$

29 $$\begin{array}{@{}rcl@{}} w_{i} \leq w_{S} && \quad \forall i \in S, \forall S \subset \{1,2,\dots,n\}, S \neq \emptyset \end{array} $$

30 $$\begin{array}{@{}rcl@{}} \sum\limits_{j=1}^{i-1} z_{ji} + \sum\limits_{j=i+1}^{n} z_{ij} = 2 w_{i} && \quad \forall i \in \{1,2,\dots,n\} \end{array} $$

31 $$\begin{array}{@{}rcl@{}} \sum\limits_{i \in S} \sum\limits_{\underset{j>i}{j \notin S}} z_{ij}+ \sum\limits_{i \in S} \sum\limits_{\underset{j<i}{j \notin S}} z_{ji} \geq 2 (w_{S} + w_{\overline{S}} -1) && \quad \forall S \subset \{1,2,\dots,n\}, S \neq \emptyset \end{array} $$

32 $$\begin{array}{@{}rcl@{}} d_{ij} \geq |S^{i}_{u} - S^{j}_{u}| && \quad \forall u, \forall i, \forall j\ \text{ such that }\ i<j\\ {\kern2.5cm} CAC \left(S^{i}_{u}, y^{i}_{r} \right) && \quad \forall i\\ {\kern2.5cm}REACH \left(S^{i}_{u}, X^{i}_{\alpha p} \right) && \quad \forall i \\ {\kern2.5cm}INC \left(X^{i}_{\alpha p} \right) && \quad \forall i \\ {\kern2.5cm}FEAS \left(X^{i}_{\alpha p}, Y^{i}_{\ell} \right) && \quad \forall i \\ {\kern2.5cm}Y^{i}_{\ell} \in \{0,1\} && \quad \forall i \in \{1,2,\dots,n\}, \forall \ell \\ {\kern2.5cm} w_{i} \in \{0,1\} && \quad \forall i \in \{1,2,\dots,n\} \\ {\kern2.5cm} w_{S} \in \{0,1\} && \quad \forall S \subset \{1,2,\dots,n\}, S \neq \emptyset \\ {\kern2.5cm} z_{ij} \in \{0,1\} && \quad \forall i, \forall j\ \text{ such that }\ i<j \\ {\kern2.5cm} d_{ij} \geq 0 && \quad \forall i, \forall j\ \text{ such that }\ i<j \\ {\kern2.5cm} S^{i}_{u} \geq 0 && \quad \forall i, \forall u \\ {\kern2.5cm} y^{i}_{r} \in \{0,1\} && \quad \forall i, \forall r \\ {\kern2.5cm} X^{i}_{\alpha p} \in \{0,1\} && \quad \forall i, \forall (\alpha,p) \end{array} $$

This model is a mixed integer program with linear constraints and a nonlinear objective function. It can be linearized by introducing the nonnegative real variables *D*_*ij*_, where *D*_*ij*_ stands for the product *d*_*ij*_ *z*_*ij*_ for each *i* and *j* with *i*<*j*. After replacing the objective function by $\sum _{i=1}^{n-1} \sum _{j=i+1}^{n} D_{ij}$ and adding the constraints *D*_*ij*_≥*d*_*ij*_−*M*(1−*z*_*ij*_) (where *M* is a constant larger than any distance between two configurations), we obtain a model of our problem that is actually a mixed integer linear program.

We expect that *n* (the maximum number of configurations) will be relatively small for most data sets, i.e., smaller than 30. If the number of tests is large, however, the above MILP will be hard to solve and one will have to investigate the use of column generation or other decomposition techniques for solving our problem. We also note that the set of solutions of this model exhibits many symmetries. This difficulty could be partially overcome by introducing the constraints *w*_*i*_≥*w*_*i*+1_ (for *i* in {1,2,…,*n*−1}) into the model.

## Results and discussion

In this section we present results pertaining to the solution of the covering and sequencing subproblems. As mentioned in the subsection “Methods”, the global formulation cannot be used for large data sets, particularly the kind of data sets that the Company must handle. We start by describing the format of each data set, consisting of a *probe file*, a *point file*, and a *test file*. The probe file contains the description of each probe, namely: a numerical identifier; the string “top” or “bot” to indicate whether the shuttle to which the probe is attached is on top or on bottom of the board; a string indicating the position of that shuttle (for instance “fl” refers to a front-left shuttle); and the coordinates of the point of attachment of the probe with respect to the preferred corner of the shuttle to which it is attached. Here is an example of such a description.

0 top fl 65.0 79.0

This means that Probe 0 is attached to the front-left shuttle on top of the board and that it is attached to this shuttle at the point of coordinates (65.0,79.0) (since the preferred corner of a front-left shuttle is the point of coordinates (0.0,0.0)). The point file contains the description of each point, i.e., a numerical identifier and the coordinates of that point. For example the line corresponding to Point 0 is the following.

0 525.0 425.0

The test file consists of a sequence of test descriptions (each of which occupying several lines). For instance the description of Test 4908 is as follows.


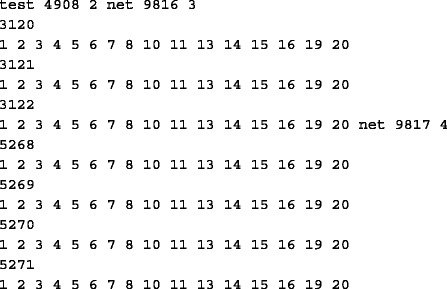


Note that the second number on the first line represents the number of nets within the test (2, in this example). Therefore the first line in the test description is followed by the description of the first net, Net 9816, followed by the description of the second one, Net 9817. Net 9816 consists of three points, represented by their identifiers and whose coordinates are stored in the point file. The points in question are Points 3120, 3121, and 3122, and each point identifier is followed (on the next line) by the list of probes that may touch that particular point. Naturally Net 9817 is described in a similar fashion. Table 1 summarizes the characteristics of the four instances that we used for our tests. It turns out that some of the tests in each of the four data sets are infeasible, i.e., there are tests that no configuration can “cover.” For this reason our program begins by determining which tests are infeasible through solving a series of partial covering problems (each with a data set consisting of one test). Then we solve the partial covering problem (see section “Methods”) for the remaining tests, each of which is feasible. Note that we did not include into the model the tests that consist of 5 nets or more, because the corresponding feasibility constraints (see the subsection “Methods”) are too numerous. In any case only Dataset 3 contains such tests and there are only 6 of them.

**Table 1:** Characteristics of the instances

Instance identifier	Nb. of probes	Nb. of points	Nb. of tests	Nb. of nets
Dataset3	21	1034	2035	4388
Dataset4	21	7445	9832	19664
7700FC	21	3016	4423	10996
pinPCB_15mils	21	6659	3331	6659

In our experiments we solved the covering problem by using Cplex to find a solution of each of the partial covering problems. Note that Cplex implements a branch-and-bound algorithm for solving mixed integer linear programming problems (see Nemhauser and Wolsey [16] for a description of the branch-and-bound method). Our initial intention was to solve the covering problem by solving partial covering problems until all the tests had been covered, as explained in the subsection “Methods”. Unfortunately the number of constraints of our model is huge and including into it all the feasible tests consumed too much memory. Therefore instead of solving a partial covering problem including all the feasible tests, we included at most 400 tests in each of the successive partial covering problems solved by our algorithm. The configuration obtained after solving a given partial covering problem may actually cover more than the number of covered tests indicated in the solution; therefore we look for all the tests covered by the current configuration before solving the next partial covering problem.

The mixed integer linear program (MILP) corresponding to a partial covering problem has a weak linear relaxation and many of its solutions have the same objective function value. Hence finding a (provably) optimal solution of a given partial covering problem takes too much time and we decided to put a limit on its execution time. For each of the four data sets, we tried three such limits: 90 seconds, 180 seconds, and 270 seconds, respectively. For instance, in Table 2, the identifier 7700FC-180 refers to an experiment with data set 7700FC in which we let the Cplex MILP algorithm run for at most 180 seconds when attempting to solve a partial covering problem. We ran our tests on a machine with an Intel Core i7-860, 2.80GHz, and a 2G memory.

**Table 2:** Results. *Real time* represents the time (in minutes) needed to solve the covering problem, *User time* the time (in minutes) consumed by all the processors, *Nb. of config.* the number of partial covering problems that were solved, and *Sol. value* the optimal value of the sequencing problem described in the subsection “Methods”

Instance	Nb. feasible tests	Real time	User time	Nb. of config.	Sol. value
Dataset3-90	1404	65	192	28	12531.5
Dataset3-180	1404	80	281	24	12927.5
Dataset3-270	1404	116	387	26	11370.3
Dataset4-90	9813	57	141	17	6887.0
Dataset4-180	9813	62	188	14	7616.6
Dataset4-270	9813	81	292	15	7016.7
7700FC-90	2855	89	139	20	9953.6
7700FC-180	2855	104	185	17	8347.6
7700FC-270	2855	77	206	15	6844.7
pinPCB_15mils-90	2360	60	103	13	6178.8
pinPCB_15mils-180	2360	65	164	11	5301.2
pinPCB_15mils-270	2360	81	261	11	5284.0

Intuitively, if we spend more time finding a good solution for each of the partial covering problems, we should be able to cover all the tests with fewer configurations and hence obtain a better solution of the planning problem. With one exception (Dataset4-90), the results in Table 2 show that spending 270 seconds to solve each partial covering problem always yields a solution that is better (i.e., of smaller length) than those obtained after spending only 90 or 180 seconds on each partial covering problem. This is true even if spending 90 or 180 seconds on each partial covering problem yields a solution with fewer configurations. Overall the results are very encouraging, because the system currently in use at the Company produces plans that may include hundreds of configurations while our solutions include fewer than 30 configurations. The real time consumed for computing a solution is considered reasonable by the Company, since the time needed to test a single board is itself substantial. Therefore it is feasible to spend one hour or so preparing the sequence of configurations that will be used for testing a specific board.

## Conclusion

In this article we have shown that the use of modelling and mathematical programming yields solutions of the test planning problem that are much better than those currently used by the Company. The new solutions can be obtained within times that are reasonable. Our results indicate that the system currently used by the Company could be replaced by a system based on mathematical programming. More work remains to be done, however, in order to strenghten the formulation of the partial covering problem. In particular we would like to find new families of cutting planes, whose introduction into the model will speed up the solution process and thus yield better solutions of the partial covering problem. Another avenue is to design heuristics (not making use of MILP models) in order to cover a large number of tests with few configurations. One could then use the MILP model described in section “Methods” to cover the remaining tests, presumably those complicated tests that include three or more nets. These improvements may enable us to find solutions of the planning problem that are as good as those reported in this article but take less time to compute. As for the model in the subsection “Methods”, we could test it on small data sets and then look for more sophisticated approaches for solving it, in the hope of computing solutions for real-world problems within reasonable times.
